# Long-term exhaustion of the inbreeding load in *Drosophila melanogaster*

**DOI:** 10.1038/s41437-021-00464-3

**Published:** 2021-08-16

**Authors:** Noelia Pérez-Pereira, Ramón Pouso, Ana Rus, Ana Vilas, Eugenio López-Cortegano, Aurora García-Dorado, Humberto Quesada, Armando Caballero

**Affiliations:** 1 Centro de Investigación Mariña, Universidade de Vigo, Facultade de Bioloxía, Vigo, Spain; 2 Facultad de Ciencias Biológicas, Departamento de Genética, Universidad Complutense, Madrid, Spain; 3 Present Address: Institute of Evolutionary Biology, School of Biological Sciences, University of Edinburgh, Edinburgh, United Kingdom

**Keywords:** Quantitative trait, Inbreeding

## Abstract

Inbreeding depression, the decline in fitness of inbred individuals, is a ubiquitous phenomenon of great relevance in evolutionary biology and in the fields of animal and plant breeding and conservation. Inbreeding depression is due to the expression of recessive deleterious alleles that are concealed in heterozygous state in noninbred individuals, the so-called inbreeding load. Genetic purging reduces inbreeding depression by removing these alleles when expressed in homozygosis due to inbreeding. It is generally thought that fast inbreeding (such as that generated by full-sib mating lines) removes only highly deleterious recessive alleles, while slow inbreeding can also remove mildly deleterious ones. However, a question remains regarding which proportion of the inbreeding load can be removed by purging under slow inbreeding in moderately large populations. We report results of two long-term slow inbreeding *Drosophila* experiments (125–234 generations), each using a large population and a number of derived lines with effective sizes about 1000 and 50, respectively. The inbreeding load was virtually exhausted after more than one hundred generations in large populations and between a few tens and over one hundred generations in the lines. This result is not expected from genetic drift alone, and is in agreement with the theoretical purging predictions. Computer simulations suggest that these results are consistent with a model of relatively few deleterious mutations of large homozygous effects and partially recessive gene action.

## Introduction

Since Darwin’s (1877) early experiments on plants, inbreeding depression, the reduction of fitness observed in inbred individuals compared to noninbred ones, has received increasing attention from researchers in multiple areas, from evolutionary, population, or conservation genetics (Charlesworth and Willis 2009; Charlesworth and Charlesworth 2010; Frankham et al. 2010), to new arising areas such as multitrophic interactions or community ecology (Kariyat and Stephenson 2019). Inbreeding depression and the reduction of population size are closely related and can lead the population to the “extinction vortex” (Gilpin and Soule 1986), which can ultimately lead to the extinction of populations and metapopulations (Frankham 2005; Wright et al. 2008; Robert 2011; Nonaka et al. 2019). The source of inbreeding depression is the inbreeding load, i.e., the component of the deleterious genetic load that is concealed in heterozygous state in outbred populations. This load is mainly ascribed to deleterious mutations with different degrees of recessivity, the contribution of overdominant fitness effects being most likely small according to empirical evidence (Hedrick 2012; Thurman and Barrett 2016). Reduced population size causes increased inbreeding and, therefore, exposes as homozygotes recessive deleterious components leading to a reduction in fitness (Roff 2002; Charlesworth and Willis 2009; Bozzuto et al. 2019), with a consequent further reduction in population size and an increase in the probability of extinction (Tanaka 1997, 2000; O’Grady et al. 2004). Finally, epistasis can also contribute to inbreeding depression by enhancing the effects of homozygosity at different loci, and can be detected by fast inbreeding experiments (see, e.g., Domínguez-García et al. 2019).

Despite the frequent evidence of inbreeding depression in nature, examples can be found in which a reduced population size and high levels of inbreeding do not translate into significant inbreeding depression (e.g., Duarte et al. 2003; Laws and Jamieson 2011; Mullarkey et al. 2013; Lobo et al. 2015; Peer and Taborsky 2005; Runemark et al. 2013; Tien et al. 2015; Caballero and Criscione 2019). This phenomenon is often explained by the purging of the inbreeding load through the action of natural selection under inbreeding (see, e.g., Hedrick and García-Dorado 2016). It is well known, both theoretically and empirically, that genetic purging can be particularly effective in eliminating lethal or severe effect deleterious mutations but also mutations of moderate effect (Hedrick 1994; Wang et al. 1999; Swindell and Bouzat 2006a; Ávila et al. 2010; Pekkala et al. 2012; García-Dorado 2012; Bersabé and García-Dorado 2013; López-Cortegano et al. 2016). The efficiency of purging to eliminate deleterious mutations depends on the inbreeding rate, which is inversely proportional to the effective population size (*N*_*e*_) and also depends on the breeding system (Glémin 2003). Purging also depends on the magnitude of the deleterious effects, being efficient against alleles with *N*_*e*_*d* > 1, where *d* is the purging coefficient, i.e., a measure of the magnitude of the deleterious recessive component of mutations masked in heterozygotes (García-Dorado 2012, 2015). The value of *d* is also the dominance effect defined as the value of the heterozygote deviated from the average of the two homozygotes (Caballero 2020, p. 44). For values of *N*_*e*_*d* below one, genetic drift overwhelms selection. According to theoretical predictions, fast inbreeding occurring in populations of very small size (e.g. full-sib lines) leads to only (or mostly) the purging of severely deleterious or lethal mutations (Hedrick 1994; Frankham et al. 2001), while slow inbreeding under large panmictic populations (say *N*_*e*_ > 20 individuals) offers more opportunities to purge weak deleterious alleles before the population reaches a large level of inbreeding, although its consequences appear later in time (Wang et al. 1999; García-Dorado 2012, 2015). Given the environment-dependent expression of the inbreeding load (Kristensen et al. 2008; Cheptou and Donohue 2011; Reed et al. 2012; Pemberton et al. 2017), purging could also be affected by environmental factors, being more effective under stable (Bijlsma et al. 1999) and competitive conditions (López-Cortegano et al. 2016).

Given the nature of purging and the multiple factors influencing its effectiveness, its detection in experimental studies is often a difficult task, especially if it is obscured by other processes. For example, adaptation in the wild or the laboratory environment may emulate the effects of purging (Crnokrak and Barrett 2002; Gilligan and Frankham 2003), and relaxation of selection in conservation programs may reduce its effects (Ballou 1997; Boakes et al. 2007; Caballero et al. 2017). The experimental studies addressing genetic purging include self-fertilization in plants (e.g. Willis 1999; Baldwin and Schoen 2019; Barrett and Charlesworth 1991) or animals (Chelo et al. 2019), forced matings between closely related individuals (such as sib matings) (e.g., Frankham et al. 2001; Reed et al. 2003; Swindell and Bouzat 2006b; Kristensen et al. 2008; Fox et al. 2008; Ávila et al. 2010; Noël et al. 2019) or random mating in small or moderate size populations (e.g., Latter et al. 1995; Reed et al. 2003; Meffert et al. 2006; Swindell and Bouzat 2006a, 2006b; Larsen et al. 2011; Pekkala et al. 2012, 2014; Bersabé and García‐Dorado 2013). A major factor hampering the detection and description of the effects of purging is, however, the elapsed time period of inbreeding. While purging can take considerable time to become visible, especially for nonlethal alleles, most purging detection-oriented experiments are limited to a small number of generations, generally not more than 20–30 (with some exceptions, such as those of Latter et al. 1995; Reed et al. 2003; Ávila et al. 2010; Chelo et al. 2019; Noël et al. 2019). Thus, a major question arises as to what are the long-term consequences of inbreeding and purging.

In a previous, long-term analysis, López-Cortegano et al. (2016) conducted experiments with two populations of *Drosophila melanogaster* in order to detect purging and quantify its magnitude. Two large populations from Madrid and Vigo labs (with census sizes of *N* ≈ 2600 and 3000 individuals, respectively) were maintained in 32 and 30 bottles, respectively, with circular mixing for more than 100 generations. In addition, multiple lines of reduced, but moderately large size (*N* = 80 and 100, respectively), were derived from the large ones and maintained for about 40 generations. The analysis reported an estimate of the overall purging coefficient of about *d* ≈ 0.3 (or 0.2 for nonlethal mutations). It also showed that purging is rather efficient in reducing the inbreeding load. Thus, over the one-hundred generation period, an initial inbreeding load for pupae productivity of about 1.8 lethal equivalents in the large Vigo population was reduced down to 0.60, and that of the Madrid population (about 2) down to 0.85, a reduction much more drastic than expected from genetic drift alone.

Although these experiments proved the efficiency of long-term purging, a substantial amount of inbreeding load still remained by the end of the period considered. According to theory, even if all the ancestral inbreeding load was removed (due to random fixation and/or purging), new inbreeding load is continuously introduced through new mutation as the populations approach a new mutation–selection–drift balance (García-Dorado 2007, 2012). However, if the current population size is small, that new balance could harbor very small inbreeding load. Thus, a question arises as to whether continuous purging can render a population of relatively large size with negligible inbreeding depression and inbreeding load, as found in some natural populations. To respond to this question, we continued the maintenance of the Madrid population and its derived lines, and we can now report inbreeding load estimates for a time span of 10 years. Our results show that the inbreeding load has been fully depleted in both the large populations and the derived lines. The evolution of the inbreeding load of the populations is compared with theoretical predictions assuming purging and genetic drift, or only this latter. We also explore the fit between the observed values and computer simulation results using a range of deleterious mutation models.

## Material and methods

### Maintenance of Drosophila populations

The large Madrid and Vigo populations (base populations founded in 2009 and 2006, respectively) had effective population sizes of approximately *N*_*e*_ ≈ 1376 and 1000, respectively, and the 64 and 20 lines derived from them, each with 80 and 100 individuals, had average *N*_*e*_ ≈ 43 (range 42–45) and 52 (range 34–63), respectively, estimated from microsatellite markers (López-Cortegano et al. 2016). These lines were established at generations 83 (Madrid) and 86 (Vigo) of the base populations. Vigo base population and lines were accidentally lost after generation 140 (a sudden electric breakdown in the flies’ culture chamber). We report here late results for these Vigo populations not included in the analyses of López-Cortegano et al. (2016), corresponding to generation 125 of the base population and generations 25 and 39 of the derived lines (this last being contemporaneous with generation 125 of the base population). Madrid base population and lines were transferred to Vigo´s laboratory at generation 129 of the base population and generation 48 of the lines (year 2015), where they were maintained thereafter. Here we report results up to generation 234 for the base population and generation 153 for the derived lines, which are contemporaneous.

Throughout the experiments, the populations were maintained in the same conditions, in a room chamber with constant temperature (25 °C) and permanent lighting, except when handling flies. The base populations were maintained in 32 (Madrid) and 30 (Vigo) bottles with circular mixing, under which, for each generation (every 2 weeks), each bottle *i* was founded with ~40–50 flies from the offspring of bottle *i* plus 40–50 flies from the offspring of bottle *i* + 1. The derived lines were maintained synchronously to the base populations in individual bottles with 80 (Madrid lines) or 100 (Vigo lines) individuals (half of each sex) per generation. Each bottle (~5 cm of diameter) was filled with ~2 cm of agar–yeast–flour–sugar medium and propionic acid (5 ml per liter of medium) to prevent fungal contamination. Only one of the Madrid lines was lost during the experiment.

### Evaluation of fitness and inbreeding load

We evaluated fitness by measuring pupae productivity (*P*) estimated as the average number of pupae produced 11 days after mating. This trait includes mating success, fecundity, and pupae survival, thus being a proxy for fitness in moderately competitive conditions. Pupae productivity and its inbreeding load (*δ*) was evaluated at generations 201 and 234 (Madrid base population) and, synchronously, at generations 120 and 153 of the derived lines. Here we add to the previously published data on the decline of the productivity mean and inbreeding load (López-Cortegano et al. 2016), results obtained at generation 125 (Vigo base population) and generations 25 and 39 (derived lines). All evaluations were conducted in the same way. At a given generation *t*, seven virgin females and seven males were sampled from each bottle and placed in pairs in individual mating vials. For the base population, males were first randomized so that they mated females from random bottles, while for the lines, the pairs were formed from flies born in the same bottle (i.e., from the same line). The offspring was used to generate two schemes: an inbred one, in which full-sib couples were mated in individual vials, and a noninbred scheme, in which a male from vial *i* was mated with a female from vial *i* + 1. At the next generation, we repeated both schemes, so that in the inbred scheme, parents had an expected inbreeding coefficient of *F* = 0.25 and their offspring *F* = 0.375 relative to the generation *t* of their population or line. In the noninbred scheme, a male from vial *i* was mated with a female from vial *i* + 2 to avoid new inbreeding, so that the corresponding expected inbreeding coefficient of both parents and their progeny was *F* = 0.

Productivity was estimated in the noninbred (*P*_*O*_) and inbred (*P*_*I*_) schemes and the inbreeding load was estimated as $\delta = \left[ {{{{{{\mathrm{ln}}}}}}\left( {P_O/P_I} \right)} \right]/F$, where *F* is the average inbreeding coefficient of parental and progeny generations, as the productivity is a trait that depends on both parental and progeny genotypes (Ávila et al. 2013). Bootstrap errors were obtained for each estimate of *δ* using the function “sample” in R. For each estimate, 1000 samples of the same size as the original productivity data (inbred and noninbred) were sampled with replacement. For each sample, *δ* was obtained as above and the standard deviation of the 1000 measures was calculated.

### Prediction of the long-term evolution of fitness and inbreeding load

The Inbreeding–Purging (IP) model (García-Dorado 2012) describes the evolution of fitness and of the inbreeding load in populations undergoing inbreeding and, therefore, exposed to the action of genetic purging. This model establishes a purged inbreeding coefficient, *g*, that equals the classical Wright’s inbreeding coefficient *F* but corrected for the expected reduction in frequency of fully or partially recessive deleterious alleles due to purging. The value of *g* can be calculated for each generation as

1 $$g_t = \left[ {\frac{1}{{2N_e}} + \left( {1 - \frac{1}{{2N_e}}} \right)g_{t - 1}} \right]\left( {1 - 2dF_{t - 1}} \right),$$

where *N*_*e*_ refers to the effective population size, *F* to Wright’s inbreeding coefficient *F*_*t*_ = (1 – [1 – (1/2*N*_*e*_)]^*t*^) and *d* to the purging coefficient. For each locus, *d* equals the recessive component of deleterious mutations that remains hidden in heterozygosis but is expressed in homozygosis. For a single locus *d* amounts to *s*(½ – *h*), where *s* represents the homozygous deleterious effect and *h* the dominance coefficient. As generations proceed, *g*_*t*_ approaches a value smaller than one, corresponding to an asymptotic situation where all the ancestral inbreeding load has been lost due to the combined effect of genetic drift and purging. The larger is *d*, the smaller are the asymptotic *g* value and the role of drift, and the fewer deleterious alleles become fixed. For *d* = 0, *g*_*t*_ reduces to *F*_*t*_. For a multilocus model with variable effects, an effective purging coefficient is defined as the value of *d* that provides the best fit between the theoretical predictions and the observed temporal evolution of the inbreeding load or the average fitness (García-Dorado et al. 2016). For pupae productivity, this *d* parameter was estimated from the evolution of the inbreeding load in the data analyzed by López-Cortegano et al. (2016) using minimum square fitting, giving a global value of *d* = 0.3 (with 95% confidence interval 0.28–0.33).

Assuming absence of purging (only genetic drift), the evolution of productivity at generation *t* expected from the inbreeding depression can be predicted as

2 $$E\left[ {P_t} \right] = E\left[ {P_0} \right] \cdot e^{ - \delta F_t},$$

where *E* stands for expectation and *δ* is the rate of inbreeding depression that, in the absence of selection, should equate the initial inbreeding load. The corresponding evolution of the inbreeding load, ignoring the contribution of new mutation, can be predicted as

3 $$E\left[ {\delta _t} \right] = \delta \left( {1 - F_t} \right).$$

However, under the IP model, the expected evolution of productivity is predicted as

4 $$E\left[ {P_t} \right] = E\left[ {P_0} \right] \cdot e^{ - \delta g_t},$$

which accounts for both inbreeding and purging, while that of the inbreeding load is

5 $$E\left[ {\delta _t} \right] = \delta g_t\left( {1 - F_t} \right)/F_t$$

which accounts both for genetic drift and purging. Note that each of these two predictions is a function of *d* and *N*_*e*_ (the main parameters determining purging and drift, respectively), which determine *F*_*t*_ and *g*_*t*_.

Taking *d* = 0 in Eq. (1) *g*_*t*_ reduces to *F*_*t*_, so that Eqs. (4) and (5) reduce to (2) and (3), respectively. Therefore, to disentangle the inbreeding load removed by genetic purging from that removed by random fixation and loss of deleterious alleles (genetic drift), the predictions of Eqs. (3) and (5) should be compared. Assuming this IP model and making use of the estimates of *N*_*e*_, *d*, and *δ* obtained by López-Cortegano et al. (2016), we predicted the expected evolution of productivity and inbreeding load over generations, and compared them with the observed results. To compute these predictions, we calculated *F* for both the base populations and the lines from the corresponding estimated *N*_*e*_ values, and subsequently we obtained *g* applying Eq. (1). From this point, *P*_*t*_ and *δ*_*t*_ were predicted considering, either a neutral model without purging (i.e., using *d* = 0 in Eq. (1), which gives *g*_*t*_ = *F*_*t*_) or a model assuming both drift and purging (using the *d* estimates in Eq. (1)).

### Computer simulations

Computer simulations were performed with an in-house C program in order to describe the range of mutational effects and dominance coefficient that better explain the evolution of fitness and inbreeding depression predicted by the model using our *d* estimate. Starting from a very large population (*N*_*e*_ = *N* = 10,000), emulating a natural population at the mutation–selection equilibrium, a dioecious sample of 1376 individuals with sex ratio of one to simulate the Madrid population (1000 individuals for Vigo, in parenthesis from here on), was taken and maintained with constant size during 83 (86) discrete generations under the action of selection and drift. Polygamous matings were allowed, where the probability of being selected as a parent to generate each offspring was determined by the individual fitness value (see below). At generation 83 (86), a line of reduced size, *N* = 43 (*N* = 52) was derived from the base population. This line was maintained synchronously with the base population under the same conditions for 250 generations.

To generate the natural population at the mutation–selection equilibrium, we established a total number of 9000 diploid genomic positions. Each position was ascribed to a homozygous selection coefficient *s* obtained from a gamma distribution with mean $\overline s$ and shape parameter *β* = 0.2 (values of *s* larger than 1 were redefined as *s* = 1), and to a dominance coefficient *h* obtained from a uniform distribution between 0 and e^(−*ks*)^, *k* being a constant needed to get the desired average value ($\overline h$) (Caballero and Keightley 1994; see Caballero 2020, p. 152–161). All mutations were assumed to have deleterious effect on fitness, where the fitness of the wild-type genotype, the heterozygote and the mutant homozygote are 1, 1 – *sh*, and 1 – *s*, respectively, and individual genotypic fitness values were obtained multiplicatively across loci. The deterministic expected equilibrium frequencies at the mutation–selection balance were obtained from

6 $$\widehat p = \left[ {\widehat p^2 + \widehat p\widehat q\left( {1 - sh} \right)} \right]\left( {1 - u} \right)/\left[ {1 - 2\widehat p\widehat qsh - \widehat q^2s} \right]$$

(Crow and Kimura 1970, p. 258), where $\widehat p$ and $\widehat q$ are the equilibrium frequencies of wild-type and mutant alleles, respectively, and *u* the mutation rate per locus and generation. Using these theoretical frequencies, allele copies were randomly distributed across individuals of the natural population. Individuals were then sampled from this natural population to found the base populations, which were subjected to the action of mutation, genetic drift, and selection.

After the formation of the natural populations, the simulation process that followed was repeated 100 times and the results were averaged. As a result, we obtained the average fitness and the average inbreeding load, computed as the sum over loci of *δ* = *s*(1 − 2*h*)*pq* (Morton et al. 1956) per generation for both the base populations and the derived lines. We explored a range of mutational parameters encompassing the values observed empirically, either assuming models of many mutations of small average effect ($\overline s$ = 0.01–0.03) or fewer mutations of large effect ($\overline s$ = 0.1–0.3), and average dominance coefficients ($\overline h$) ranging between 0.1 and 0.3. The mutation rate was adjusted so that the simulated populations had an initial inbreeding load close to that inferred in the experimental populations. This implied a haploid genomic mutation rate per generation of around *U* = 0.1 for the models of small-effect mutations and one of around *U* = 0.02 for the models of large-effect mutations. The mutational parameters assumed are within the range of those obtained empirically from mutation-accumulation experiments (Caballero 2020, p. 152–157). Sampled *s* values larger than 1 were assigned a value *s* = 1 so that the mutational model generates a lethal class, thus producing a bimodal distribution of selection coefficients in the case of large-effect mutations (see Supplementary Material Fig. S1). We also considered mutational estimates obtained from the evolutionary analysis of genomic data (Kim et al. 2017), to follow the conditions of the simulations carried out by Kyriazis et al. (2020). This model considers that the average homozygous selection coefficient is $\overline s$ = 0.0161, with effects obtained from a gamma distribution with shape parameter *β* = 0.186. The model assumes that the dominance coefficient is a constant value of *h* = 0.25 when the mutation homozygous effect is *s* < 0.02 and *h* = 0 otherwise. Thus, the model is similar to one of the above models of mutations with small effects except for the dominance assumed. As in the previous models, the mutation rate was adjusted to produce an initial inbreeding load close to the observed ones, giving *U* = 0.16, which is very close to the value assumed by Kyriazis et al. (2020) (*U* = 0.21). Simulations were also carried out assuming a neutral (only genetic drift) model in order to check the accuracy of neutral predictions. The fit between the simulation and observed results was quantified by the mean square difference between both values considering all generations of the base population and lines.

## Results

### The effect of long-term purging on the inbreeding load

Tables 1–2 show results for the estimates of the inbreeding load (*δ*) over generations reported by López-Cortegano et al. (2016) or obtained in the present study. For Madrid base population (Table 1), the inbreeding load dropped to about the same amount at generations 201 and 234, being nonsignificantly different from zero in the last generation (*δ* = 0.145 ± 0.098; *p* = 0.066). In the case of Madrid derived lines, inbreeding depression was largely reduced after 120 generations and virtually exhausted after 153 generations (*δ* = 0.014 ± 0.083; *p* = 0.439). Note, however, that the measures at both generations were not significantly different from each other (*p* = 0.124). For the Vigo base population (Table 2), the decline in the inbreeding load was rather linear until generation 111 (López-Cortegano et al. 2016). However, in the latest generation (Gen. 125), the inbreeding load showed a substantial drop down to a nonsignificant value (*δ* = 0.11 ± 0.07; *p* = 0.051). Regarding Vigo’s lines, the inbreeding load was slightly reduced after 25 generations but, again, was exhausted by generation 39 (*δ* = −0.05 ± 0.06; *p* = 0.755).

**Table 1:** Pupae productivity in Madrid populations.

MADRID	*F* mother	*F* offs.	*n*	Productivity ± se	*δ* ± se	Reference
Base population						
Gen. 0					2.00	Inferred IP LC2016
Gen. 112	0	0	108	86.91 ± 2.12	0.848 ± 0.142	LC2016
	0.25	0.25	200	70.30 ± 1.79		
Gen. 113	0	0	159	81.14 ± 1.99	–	LC2016
	–	–	–	–		
Gen. 201	0	0	210	94.99 ± 1.12	0.151 ± 0.070	This study
	0.25	0.375	184	90.60 ± 1.67		
Gen. 234	0	0	215	86.96 ± 1.40	0.145 ± 0.098	This study
	0.25	0.375	144	83.11 ± 2.21		
Lines						
Gen. 0 (g.83 BP)					1.402	Inferred LC2016
Gen. 30 (g.113 BP)	0	0	171	66.92 ± 2.27	–	LC2016
	–	–	–	–		
Gen. 120 (g.201 BP)	0	0	337	81.84 ± 1.25	0.141 ± 0.075	This study
	0.25	0.375	339	78.30 ± 1.43		
Gen. 153 (g.234 BP)	0	0	335	72.04 ± 1.46	0.014 ± 0.083	This study
	0.25	0.375	323	71.72 ± 1.41		

*F*: coefficient of inbreeding in mothers and offspring; *n*: number of pairs evaluated, se: standard error of productivity means, *δ*: estimate of inbreeding load with standard error (se) obtained by bootstrapping, inferred IP: values of inbreeding load inferred by the Inbreeding–Purging model (García-Dorado 2012), BP: base population, LC2016: López-Cortegano et al. (2016).

**Table 2:** Pupae productivity in Vigo populations.

VIGO	*F* mother	*F* offs.	*n*	Productivity ± se	*δ* ± se	Reference
Base population						
Gen. 0					1.85	Inferred IP LC2016
Gen. 22	0	0	71	101.97 ± 2.38	1.744 ± 0.112	LC2016
	0.375	0.5	99	47.56 ± 2.11		
Gen. 50	0	0	147	45.94 ± 1.01	1.395 ± 0.136	LC2016
	0.25	0.375	145	29.71 ± 0.96		
Gen. 103	0	0	22	95.32 ± 4.39	0.666 ± 0.188	LC2016
	0.375	0.5	38	71.24 ± 5.33		
Gen. 111^a^	0	0	227	87.30 ± 2.08	0.609 ± 0.109	LC2016
	0.25	0.375	251	72.16 ± 1.83		
Gen. 125	0	0	388	64.35 ± 0.70	0.115 ± 0.069	This study
	0.25	0.375	252	62.08 ± 1.18		
Lines						
Gen. 0 (g.86 BP)					0.92	Inferred IP LC2016
Gen. 25 (g.111 BP)	0	0	239	89.49^b^ ± 1.66	0.728 ± 0.111	This study
	0.25	0.375	230	71.28 ± 2.05		
Gen. 39 (g.125 BP)	0	0	283	58.35 ± 0.79	−0.046 ± 0.063	This study
	0.25	0.375	315	59.19 ± 0.89		

*F*: coefficient of inbreeding in mothers and offspring, *n*: number of pairs evaluated, se: standard error of productivity means, *δ*: estimate of inbreeding load with standard error (se) obtained by bootstrapping, inferred IP: values of inbreeding load inferred by the Inbreeding–Purging model (García-Dorado 2012), BP: base population, LC2016: López-Cortegano et al. (2016).

^a^Results corresponding to generation 3 of experiment A of Domínguez-García et al. (2019).

^b^Value reported by LC2016.

**Fig. 1:**
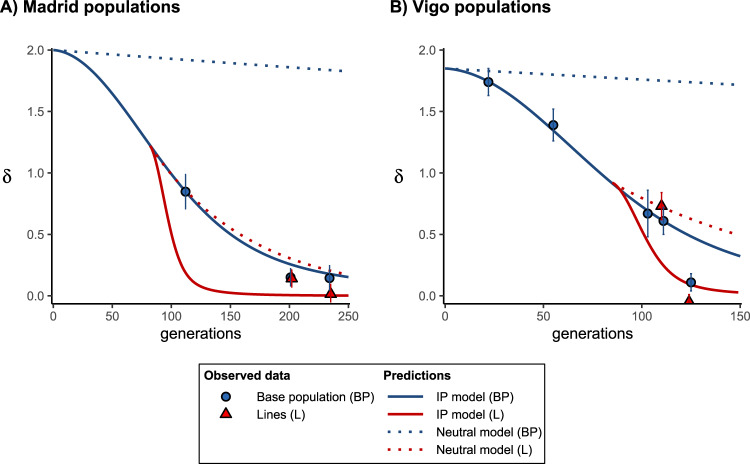
Prediction (lines) of the expected evolution of the inbreeding load (*δ*) for both the base population and the derived lines, considering the Inbreeding–Purging (IP) model (continuous lines) and a neutral one without purging (only genetic drift; dotted lines), and observed values (symbols) from Tables 1 and 2. Predictions are obtained with the empirical estimates. Bars indicate one bootstrap standard error. Generations correspond to the time scale of the base population. **A** Madrid populations; **B** Vigo populations.

Figure 1 shows the fit of the expected declines in inbreeding load under the IP model (continuous lines) and the neutral model (dotted lines) to the observations for both base populations and derived lines. The observed inbreeding load in the last generation evaluated was nonsignificantly different from the expectations under a purging model in the case of the Madrid base population (*p* = 0.392) and lines (*p* = 0.442) (Fig. 1A), but lower than the expectations in the case of the Vigo base population (*p* < 0.001) and lines (*p* = 0.011) (Fig. 1B). The inbreeding load expected under a neutral model (only genetic drift) was very much larger than that observed experimentally in all cases, except for generation 25 of Vigo’s lines.

A comparison between the productivity means across generations must be done with caution, as the cross-generational estimates are subjected to environmental fluctuations. Although the flies were maintained in a chamber with uniform and constant environmental conditions, the external environmental changes throughout the year (which could take a role during the handling of flies in the laboratory) could affect very sensitive traits such as female fecundity. The mean productivity of the Madrid base population declined from generation 201 to 234 by 8.4% in the outbred estimate and by 8.3% in the inbred one (Table 1). The corresponding declines for the Madrid lines were 12 and 8.4%. Therefore, the change in mean over generations was roughly the same for the base population and for the lines. For Vigo base population, the mean productivity dropped from generation 111 to 125 by 26% in the outbred estimate and by 14% in the inbred one, and the corresponding drops in Vigo lines were 35 and 17%. Thus, although somewhat larger in the lines, the drops were not very different between the base population and the lines. Altogether, this suggests that the drop in productivity observed between these generations was not mainly due to inbreeding depression, which should progress much faster in the lines with *N*_*e*_ ≈ 50 than in the populations with *N*_*e*_ ≈ 1000, but is most likely due to environmental causes.

**Fig. 2:**
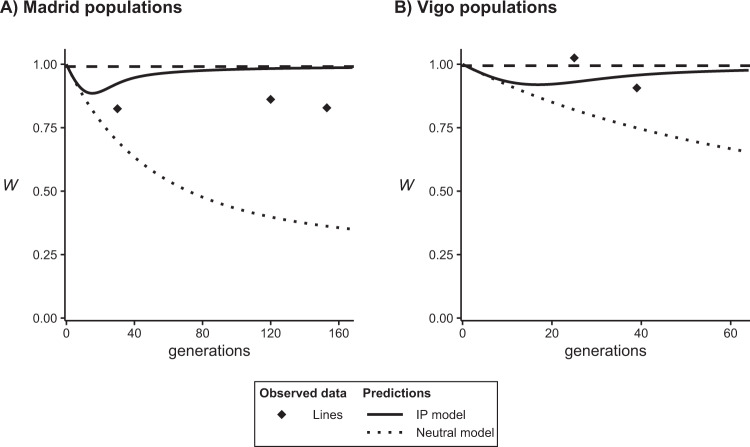
Prediction (lines) of the expected evolution of pupae productivity (*P*) in the lines (relative to that of the corresponding synchronous base population), considering the Inbreeding–Purging (IP) model (continuous lines) and a neutral one without purging (only genetic drift; dotted lines), and observed values (symbols) from Tables 1 and 2. Predictions are obtained with the empirical estimates. The horizontal dashed line indicates the theoretically maximum relative productivity. **A** Madrid populations; **B** Vigo populations.

A comparison between the estimates of mean productivity of the base population and lines in the same generation is more reliable, as they were obtained synchronously. Figure 2 presents the average productivities of the noninbred lines relative to those obtained in the corresponding base populations for which the IP model predicts negligible depression. Again, the observed results were clearly closer to the expectations under a purging model than under a neutral model, although the predictions of the purging model for the Madrid lines were consistently higher than the observed values. Thus, the expected mean productivities under the purging model in the latest generation were 20% and 5% higher than the observed values in the Madrid and Vigo lines, respectively, whereas the expectations under the neutral model were 56% and 17% lower, respectively.

### Computer simulations

**Fig. 3:**
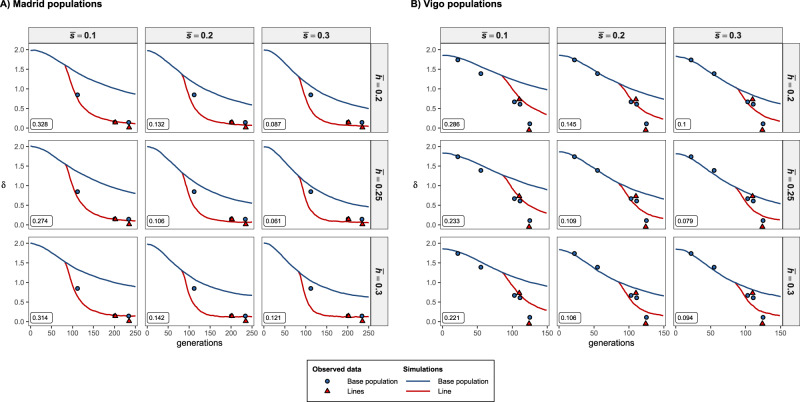
Evolution of the inbreeding load (*δ*) in simulated populations corresponding to Madrid’s (**A**) and Vigo’s (**B**) populations and averaged over 100 replicates. The mutational model assumed deleterious homozygous mutation effects obtained from a gamma distribution with shape parameter *β* = 0.2, mean $\overline s$, and average dominance coefficient $\overline h$. Observed values (symbols) from Fig. 1 are shown as a reference. The fit between simulation and observed values is shown as the mean square difference considering the values of the base population and small lines and shown in a box for each model.

**Fig. 4:**
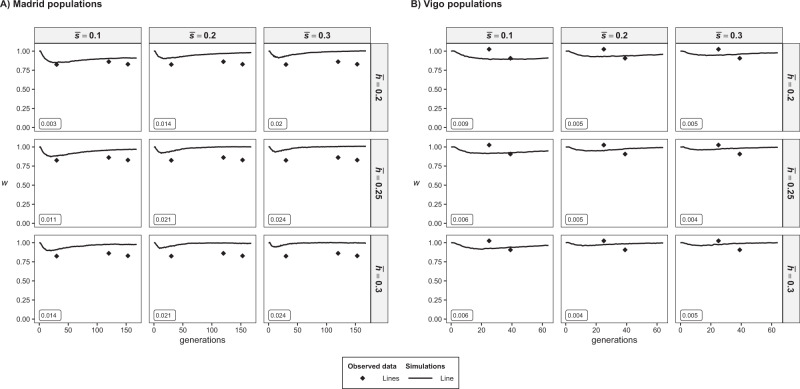
Evolution of the simulated relative fitness (*W*) corresponding to Madrid’s (**A**) and Vigo’s (**B**) populations and averaged over 100 replicates. The mutational model assumed deleterious homozygous mutation effects obtained from a gamma distribution with shape parameter *β* = 0.2, mean $\overline s$, and average dominance coefficient $\overline h$. Observed values (symbols) from Fig. 2 are shown as a reference. The fit between simulation and observed values are shown as the mean square difference considering the values of the base population and small lines and shown in a box for each model.

Computer simulations were performed in order to assess which set of mutational parameters better explain the experimentally observed inbreeding loads of base populations and derived lines and the relative fitness of the lines. All experimental results were generally consistent with simulations assuming large homozygous deleterious effects and small genomic mutation rates (*U* ≈ 0.02; Figs. 3 and 4). The average selection and dominance coefficients of mutations that better explained the inbreeding load results were $\overline s$ = 0.3 and $\overline h$ = 0.25. Simulations assuming effects tenfold smaller ($\overline s$ = 0.01–0.03) and a mutation rate five times larger (*U* ≈ 0.1) were clearly inconsistent with the empirical and theoretical predictions (Figs. S2 and S3). The same could be concluded regarding the mutation model assumed by Kyriazis et al. (2020) (Figs. S4 and S5). Simulations assuming the removal of inbreeding load only by genetic drift were very close to the neutral predictions (Figs. S6 and S7).

## Discussion

Genetic purging has long been considered an effective force in reducing the inbreeding load ascribed to lethal and large-effect mutations, particularly under fast inbreeding (Hedrick 1994; Wang et al. 1999), but its efficiency against minor mutations under slow inbreeding is less obvious (Leberg and Firmin 2008). The time needed for purging deleterious mutations of small effect imposes an important limitation when it comes to detecting it, both in experimental populations (too long experiments, difficulty in handling, etc.) and in natural populations (not enough information is usually available), as has been previously noted (Gulisija and Crow 2007; García-Dorado 2015). In two long-term Drosophila analyses under slow inbreeding, López-Cortegano et al. (2016) showed that purging was very effective in reducing the inbreeding load to a great extent, but at the latest generations, the reported populations still harbored substantial inbreeding load. In this work, we continued one of those experiments and report late unpublished results of the other in order to evaluate how far the original inbreeding load can be removed in the long term by genetic purging. The base populations, with estimated effective sizes over 1000 individuals, reached a state of slight and nonsignificant inbreeding load after 5 or 10 years under laboratory conditions, with an average of 24 generations per year. The final inbreeding load of the derived lines, maintained with an effective size around 50 for up to 153 or 125 generations, was almost negligible. In the case of the Madrid population, maintained for the longest period, the two final estimates (generations 201 and 234) suggest that the population is close to a plateau.

The above results are a sound evidence of purging, as the observed decline of the inbreeding load fits far better to Inbreeding Purging predictions than to the much faster decline predicted under a neutral (only genetic drift) model, both for the populations and for the lines (see Fig. 1). In addition, an exhaustion of the inbreeding load due to genetic drift alone would have required a much longer process and, more importantly, it would have been accompanied by a drastic decline in average fitness. It should be noted that IP predictions are computed ignoring both standard non-purging selection and new deleterious mutation. Particularly for large populations, predictions taking into account these factors should be more appropriate (see the full-model approach in García-Dorado 2012) but, unfortunately, this requires reliable estimates of too many genetic parameters. However, the very small value of the late estimates of the inbreeding load suggest that the role of new mutation in generating new inbreeding load has been rather small and that the simple Inbreeding Purging approach can provide reasonable long-term predictions even in moderately large populations.

The IP model for the average fitness of the lines gave also better predictions than the neutral one (Fig. 2), although neither of the two fittings was very good. It is worth noting the constant overestimation that showed the IP prediction in the lines from the Madrid experiment. This might be explained by inbreeding depression ascribed to deleterious alleles with too small effects to be efficiently purged (i.e., with *d* values much smaller than assumed to compute the predictions), but this should also cause a poor fitting for the inbreeding load predictions, which was not observed (Fig. 1). There was also a drop in productivity over the two last generations for both Madrid (generations 201–234) and Vigo (generations 111–125) experiments (Tables 1 and 2), but the drop was not very different for the large population (with *N*_*e*_ over 1000) and the lines (with *N*_*e*_ around 50), particularly in the Madrid experiment. This indicates that the late fitness drop cannot be explained by inbreeding depression due to inefficient purging, as this would have caused a much larger fitness drop in the small lines than in the large populations. It can neither be ascribed to new mutations with deleterious effect so small as to escape selection even in the large population, as this would require a huge mutation rate.

Other experiments have shown an exhaustion or drastic reduction of the inbreeding load, but for populations of much lower census sizes, where an important reduction would be expected from genetic drift alone. For example, Swindell and Bouzat (2006a) showed a decline of the inbreeding load in *D. melanogaster* to one third its initial value in populations maintained by mass mating ten breeding pairs for 19 generations, which represents a decline not much larger than expected from drift. Other long-term Drosophila studies with large census sizes also detected a reduced inbreeding load, but not its complete depletion. For example, Ávila et al. (2010) studied the effect of purging induced by restricted panmixia in a population of size *N* = 220 individuals (distributed among 55 vials), and reported a 44% reduction of *δ* for competitive fitness after 34 generations, and of 77% for viability after 60 generations.

Substantial reductions of the inbreeding load have also been observed under fast inbreeding (e.g., full-sib lines). This latter purges lethal and severely deleterious alleles but, contrary to slow inbreeding, usually leads to a continuous decline in fitness because of the fixation of mild or moderate deleterious mutations (e.g., Pekkala et al. 2014; Sharp and Agrawal 2016; Domínguez-García et al. 2019; see also Lynch and Walsh 1998, p. 255). For example, Domínguez-García et al. (2019) carried out a fast inbreeding experiment (5–6 generations of full-sib mating) with two Drosophila populations, one of them being the Vigo population referred to in this paper (in fact, the results of generation 111 of Table 2 correspond to those of the third generation of full-sib mating in experiment A of Domínguez-García et al. 2019). This experiment showed a continuous decline in fitness with inbreeding depression accelerated in the latest generations, suggesting synergistic epistasis among deleterious alleles. In this regard, and considering that synergistic epistasis may facilitate the joint elimination of interacting deleterious mutations (Kondrashov 1988; Kouyos et al. 2007), an interesting result found in our analysis (Table 2 and Fig. 1B) was the fast drop of the inbreeding load observed in the base population of Vigo from generation 111 to 125, a reduction not predicted by the IP model. It cannot be discarded that some synergistic epistatic interactions between deleterious alleles may have induced a late enhanced purging of these alleles, potentially observable under slow inbreeding, in disagreement with the prediction of the IP model, which ignores epistasis.

There are additional evidences of purged inbreeding load under fast inbreeding, as that provided by Chelo et al. (2019), who detected a reduced extinction risk in experimental populations of *Caenorhabditis elegans* with high selfing levels. Fox et al. (2008) assayed the reduction of the inbreeding load that could be ascribed to purging by measuring it in the outbred cross of three generations of full-sib inbred lines, and observed an important average reduction in the beetle *Stator limbatus*, implying that about half the original inbreeding load was due to severely deleterious mutations. In contrast, Willis (1999) only detected a slight reduction in the outbred cross of selfed lines of *Mimulus guttatus*, suggesting that lethals or mutations of severe effect were not the main contributors to inbreeding load in those populations. This could be explained by the continuous removal of severely deleterious alleles by selfing in *Mimulus guttatus*. Barrett and Charlesworth (1991) also showed that the genetic load present in an outcrossing population of *Eichhornia paniculata* exposed to five generations of self-fertilization could be explained only with a high mutation rate to partially recessive deleterious alleles, and that inbreeding purged these alleles from the population.

Our results show that purging under slow inbreeding is effective in removing most inbreeding load. This finding is in accordance with several previous empirical results (Day et al. 2003; Reed et al. 2003; Swindell and Bouzat 2006a; Pekkala et al. 2012, 2014). Note that purging most inbreeding load is likely to imply purging substantial load due to non-severely deleterious alleles. For example, in the simulated case with $\overline s$ = 0.2 and $\overline h$ = 0.25, about 15% of the inbreeding load is ascribed to deleterious alleles with *s* < 0.2. Other studies, however, have failed to detect a significant reduction in inbreeding depression under relatively slow inbreeding. For example, Kristensen et al. (2011) did not find a reduction in inbreeding depression in *Drosophila* populations with slow inbreeding (*N*_*e*_ = 32 during 19 generations) compared to populations with fast inbreeding (one generation of full-sib mating), and Leberg and Firmin (2008) did not find evidences of purging on mosquitofish populations after serial bottlenecks (consisting of a reduction of the population size to 5 or less individuals, followed by an expansion of up to 300 individuals). These contrasting results could be ascribed to the small experimental scale in terms of generation numbers.

Our experimental results show the drastic long-term effect of genetic purging in removing the initial inbreeding load for moderately competitive fitness of populations maintained in the laboratory, and we may wonder how far our conclusions could be extrapolated to the wild. The expression and severity of the inbreeding depression is environment-dependent, often being more pronounced in harsher environments (Martin and Lenormand 2006). Such interaction may be the result of a differential expression of phenotypes under selection (plasticity), an environment-dependent dominance, or a differential selection pressure (Cheptou and Donohue 2011). Therefore, alleles that in laboratory conditions (or a particular benign environment in the wild) cannot be purged even under slow inbreeding because they show only slight or no deleterious effect, may induce substantial depression in a harsher or competitive environment. Thus, Bijlsma et al. (1999) have already noted that purging efficiency depends on the conditions under which it occurs, and that effective purging in a given environment may not prevent inbreeding depression under different conditions (see also Swindell and Bouzat 2006b). However, as shown by López-Cortegano et al. (2016), although inbreeding depression can be larger in more competitive conditions due to the larger deleterious effects, purging should also be more efficient. Furthermore, these authors observed that purging occurred in competitive conditions can also be efficient against inbreeding load expressed in noncompetitive ones, suggesting that the larger inbreeding load expressed under high competition could be mainly due to the same deleterious alleles expressed in a noncompetitive environment, but with more severe effects. Thus, our IP conclusions could be expected to hold in natural populations maintained in the wild.

The virtually complete depletion of inbreeding load by genetic purging observed in our experiments is compatible with several examples of natural populations showing little or no evidence of inbreeding depression for particular traits in populations with a history of inbreeding. For example, a reduced inbreeding load for juvenile survival was found in the bottlenecked Stewart Island robin (*Petroica australis rakiura*) population, with an estimated value of 0.24 lethal equivalents (Laws and Jamieson 2011). An absence of inbreeding depression among different life-history traits was also found in the ambrosia beetle *Xylosandrus germanus* (Peer and Taborsky 2005), and for several early fitness traits in a population of the tree *Ceiba pentandra*, with variable selfing rates among maternal trees (Lobo et al. 2015). A reduced inbreeding load (*δ* = 0.19) was observed in the tapeworm *Oochoristica javaensis* with mixed mating (Caballero and Criscione 2019) and in the case of the captive population of Cuvier’s Gazelle, which showed a positive relationship between juvenile survival and inbreeding (Moreno et al. 2015). Other examples of observed reduced or lacking inbreeding depression in wild populations include populations of the lizard *Podarcis gaigeae* (Runemark et al. 2013), the greater white-toothed shrew *Crocidura russula* (Duarte et al. 2003; *δ* = 0.3 for fecundity), and the invasive biennial *Alliaria petiolata* (Mullarkey et al. 2013).

Recent studies at the genomic level added more evidence about the action of purging against deleterious alleles. Xue et al. (2015) and Grossen et al. (2020) detected a reduction of the genomic load for mutations classified as highly deleterious in populations of mountain gorillas and *Alpine ibex*, respectively, both with moderate sizes and a history of bottlenecks, but no for putatively mildly ones. Although the true magnitude of the corresponding effects is unknown, and that for the putatively mildly alleles could in fact be very small and irrelevant in the time scale of laboratory experiments or conservation management programs, these results highlight the importance of maintaining a high population size, above 1000 individuals, to prevent the accumulation of deleterious mutations that might put in risk the long-term population survival. However, the reduction of the genomic load may not appropriately reflect the reduction in fitness inbreeding load and inbreeding depression. For example, inbreeding depression could be smaller than suggested by the genomic load due to the purging of the more severe deleterious mutations, as seems to be the case of island foxes (Robinson et al. 2018), with a higher proportion of missense and loss-of-function mutations than the mainland gray foxes but no signs of inbreeding depression (presence of congenital defects). Thus, although genomic-based information can be useful in order to assess conservation efforts and to ensure the survival of inbred populations, the main relevance of purging relies primarily on its impact on the fitness inbreeding load.

Among all the mutational models tested in the simulation analyses, a set of mutational parameters produced results that fit well the observed inbreeding load and fitness, as well as the corresponding IP predictions. These parameters are a low genomic mutation rate of *U* ≈ 0.02 per haploid genome and generation, a relatively large average deleterious effect ($\overline s$) of about 0.3, and a moderate average dominance coefficient ($\overline h$) around 0.25. These parameters are within the range of those generally found for eukaryotic species from mutation-accumulation studies (see, e.g., Caballero 2020, p. 161) including a lethal class (see distribution in Fig. S1). The good fit between the predictions obtained with this low rate of mutations of large-effect and the experimental results suggests that most mutations of tiny effect that can be classified as deleterious in terms of molecular evolution (Haag-Liautard et al. 2007), which may be relevant for evolutionary time scales, contribute little inbreeding load for relative short-time spans regarding genetic conservation or animal breeding (García-Dorado and Caballero 2000; Caballero 2020, p. 157). Simulations performed by Domínguez-García et al. (2019) in relation to fast inbreeding by full-sib mating also support this model. In both cases, a model of many more mutations (five times larger mutation rate) of small effect (ten times lower) seems incompatible with the experimental results. The same can be concluded with respect to the mutation model assumed by Kyriazis et al. (2020) (Figs. S4 and S5), which implies that mutations of moderately large effect (say with selection coefficient *s* > 0.1) are very scarce, and does not consider lethal mutations (García-Dorado and Caballero 2021). This contrasts with experimental evidence supporting that deleterious mutations of moderately large effect, as well as lethals, are common and have a substantial impact on inbreeding depression (Caballero and Keightley 1998; Bijlsma et al. 1999).

In conclusion, although large population sizes are always to be preferred in order to preserve biodiversity and avoid fixation of deleterious mutations, our results illustrate the potential of purging under slow inbreeding to remove the original inbreeding load in moderate size populations, and to lead the populations toward an equilibrium with little or no inbreeding depression. It is important to emphasize that the magnitude of the deleterious effects being purged and of the expressed inbreeding depression can be influenced by the environment, so that populations should preferably be maintained in the wild if a proper assessment of the habitat is carried out. This has important implications, not only in conservation programs (either natural or captive populations), but also in breeding programs (livestock, aquaculture, etc.), where population sizes are often small and usually face the problem of inbreeding.

## Supplementary information

Supplemental material

## Data Availability

Data and simulation programs are available at https://github.com/noeliaperezp/Long-term-purging-in-Drosophila.
