# Supplementary material for: Long-term exhaustion of the inbreeding load in Drosophila melanogaster
Source: Heredity (Edinb). 2021 Aug 16;127(4):373–83. doi: 10.1038/s41437-021-00464-3 (PMC8478893; doi:10.1038/s41437-021-00464-3)

**SUPPLEMENTAL MATERIAL**

**Long-term exhaustion of the inbreeding load in *Drosophila melanogaster***

Noelia Pérez-Pereira, Ramón Pouso, Ana Rus, Ana Vilas, Eugenio López-Cortegano, Aurora García-Dorado, Humberto Quesada and Armando Caballero

**Figure S1.** Distribution of mutational effects considered for the models of small-effect mutations (average $\bar{s}$ = 0.03) and large-effect mutations (average $\bar{s}$ = 0.3). The distributions are scaled by the assumed mutation rate (*U* = 0.1 and 0.02, respectively).

**
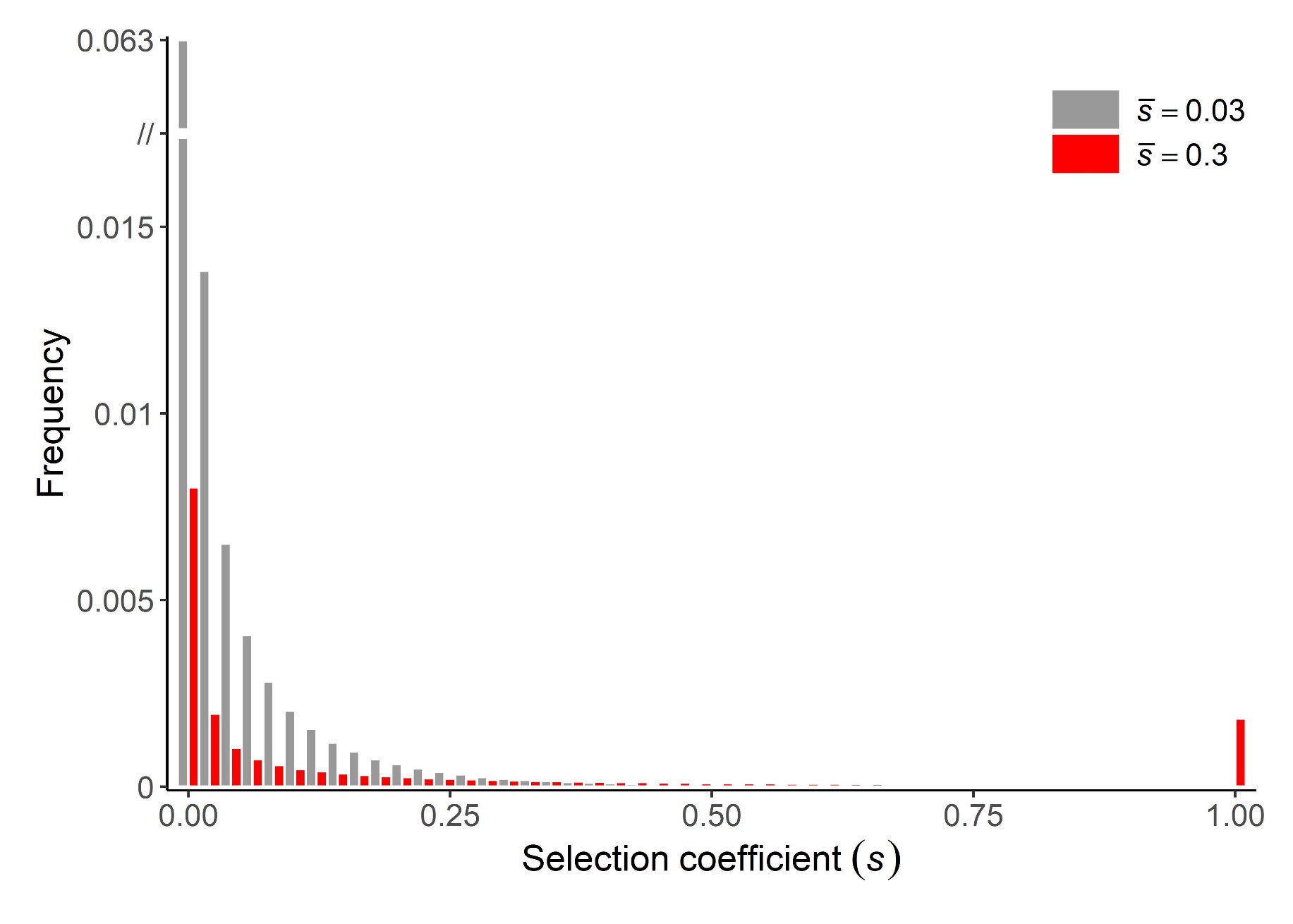
**

**Figure S2.** Evolution of the inbreeding load (*δ*) in simulated populations corresponding to Madrid´s (A) and Vigo´s (B) populations. A large base population (*N* = 1376 and 1000 for panels A and B, respectively) and a line of reduced size (*N* = 43 and 52, respectively) derived from the base population at generation 83 and 86, respectively, are simulated and the observed values averaged over 100 replicates. The mutational model assumed deleterious homozygous mutation effects obtained from a gamma distribution with mean $\bar{s}$ and shape parameter *β* = 0.2, dominance coefficients of mutations inversely related to homozygous effects (see text), and a mutation rate per haploid genome and generation fitted to obtain the approximate initial value of inbreeding load. The initial small increase in the inbreeding load observed in some simulations is due to the fact that the initial load was obtained with the infinite mutation-selection model of Eqn. 6 in the main text, that assumes recurrent mutation, whereas the simulations assume non-recurrent mutation. Observed values (symbols) from Figure 1 of the main text are shown as a reference. The fit between simulation and observed values are shown as the mean square difference considering the values of the base population and small lines and shown in a box for each model.


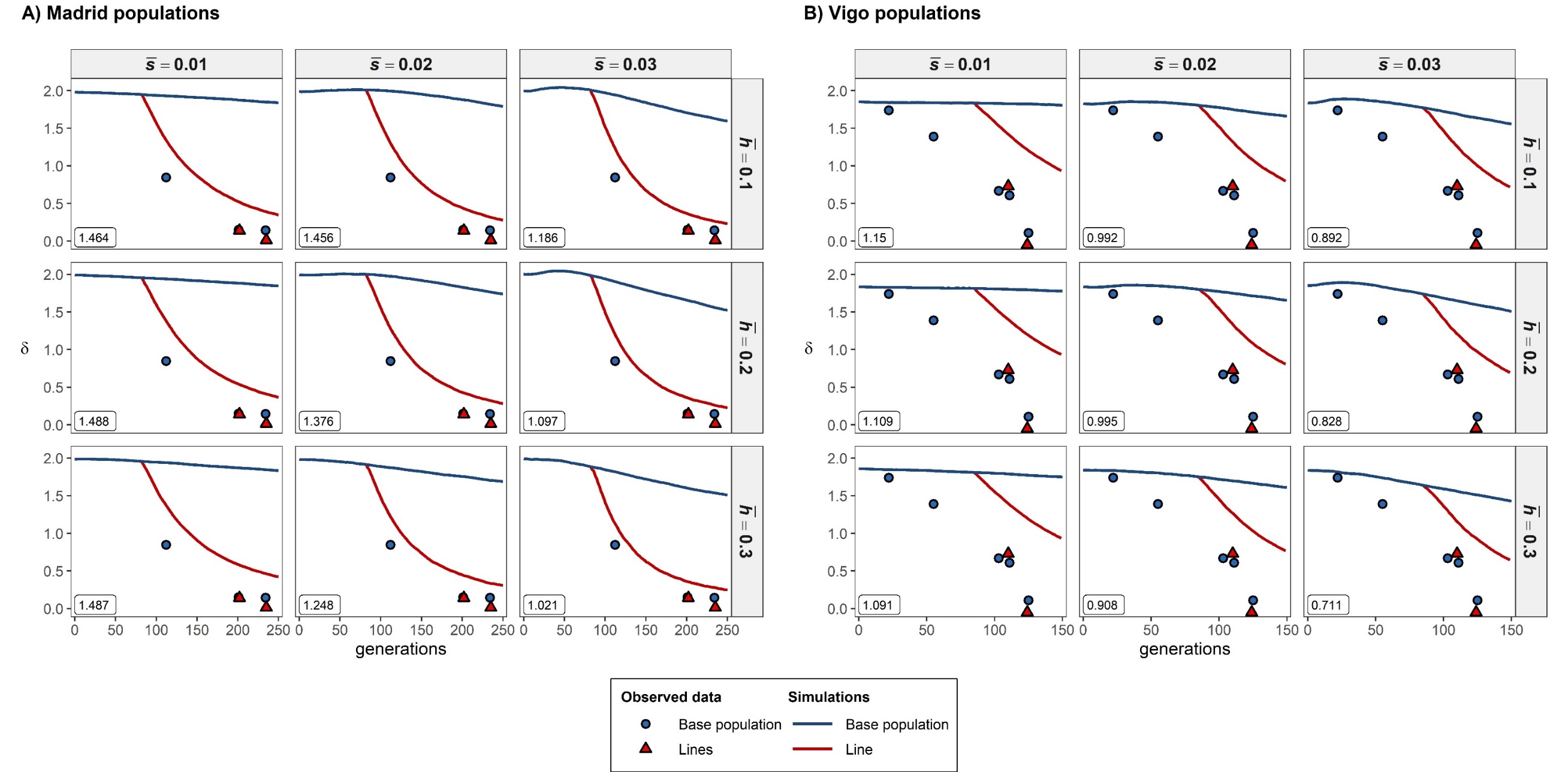


**Figure S3.** Evolution of relative fitness (*W*) corresponding to Madrid´s (A) and Vigo´s (B) populations. A simulated line of reduced size (*N* = 43 and 52 for panels A and B, respectively) derived from a base population (*N* = 1376 and 1000) after 83 and 86 generations, respectively, was run and values were averaged over 100 replicates. The mutational model assumed is described in Figure S1 legend. Observed values (symbols) from Figure 2 of the main text are shown as a reference. The fit between simulation results and observed values are shown as the mean square difference considering the values of the base population and small lines and shown in a box for each model.


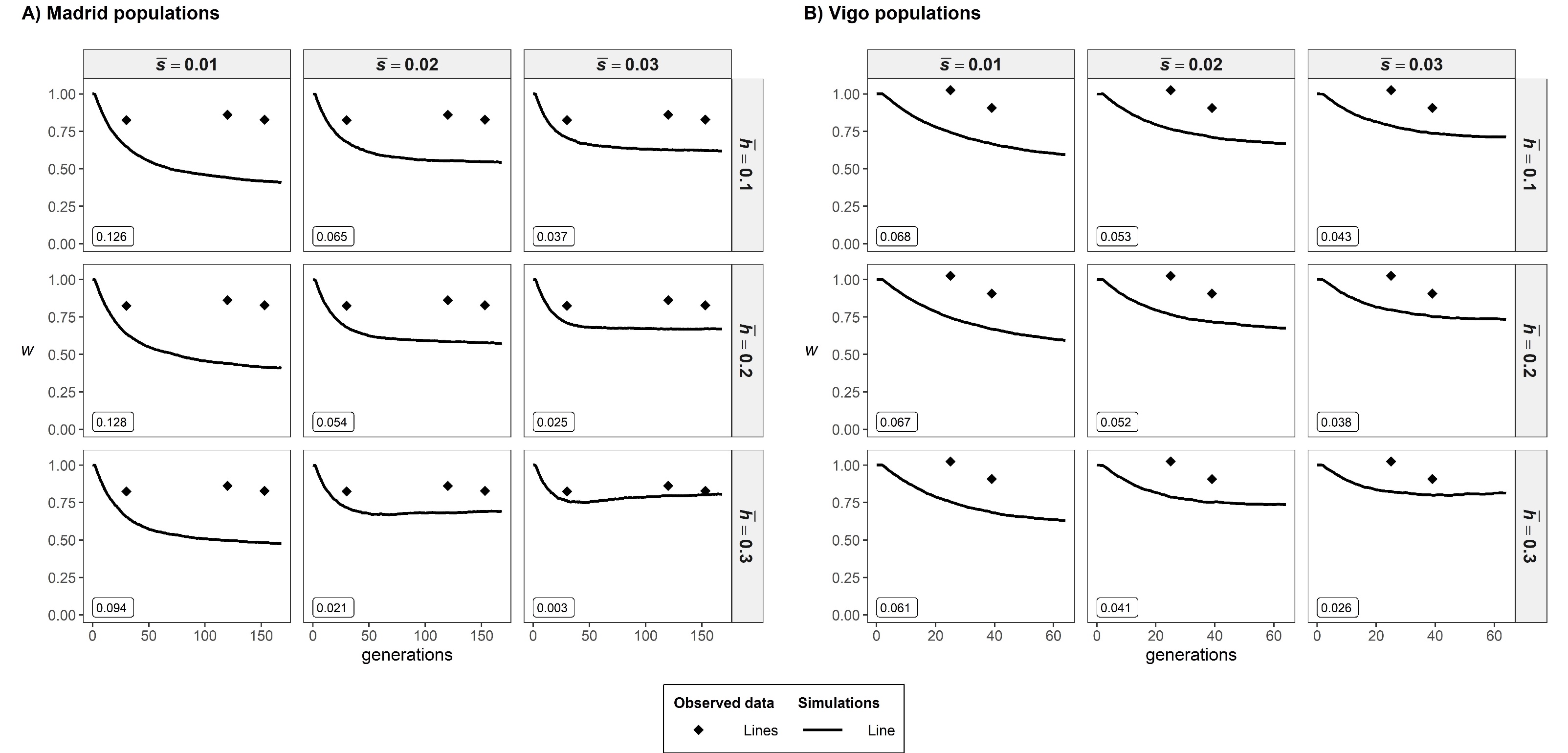


**Figure S4.** Evolution of the inbreeding load (*δ*) in simulated populations corresponding to Madrid´s (A) and Vigo´s (B) populations. A large base population (*N* = 1376 and 1000 for panels A and B, respectively) and a line of reduced size (*N* = 43 and 52, respectively) derived from the base population at generation 83 and 86, respectively, are simulated and the observed values averaged over 100 replicates. The mutational model was that assumed by Kyriazis et al. (2020), which considers deleterious homozygous mutation effects obtained from a gamma distribution with mean $\bar{s}$ = 0.0161 and shape parameter *β* = 0.186. The model assumes that the dominance coefficient is a constant value of *h* = 0.25 when the mutation homozygous effect is *s* < 0.02 and *h* = 0 otherwise. The mutation rate per haploid genome and generation was fitted to obtain the approximate initial value of inbreeding load. Observed values (symbols) from Figure 1 of the main text are shown as a reference. The fit between simulation and observed values is shown as the mean square difference (box) considering the values of the base population and small lines.


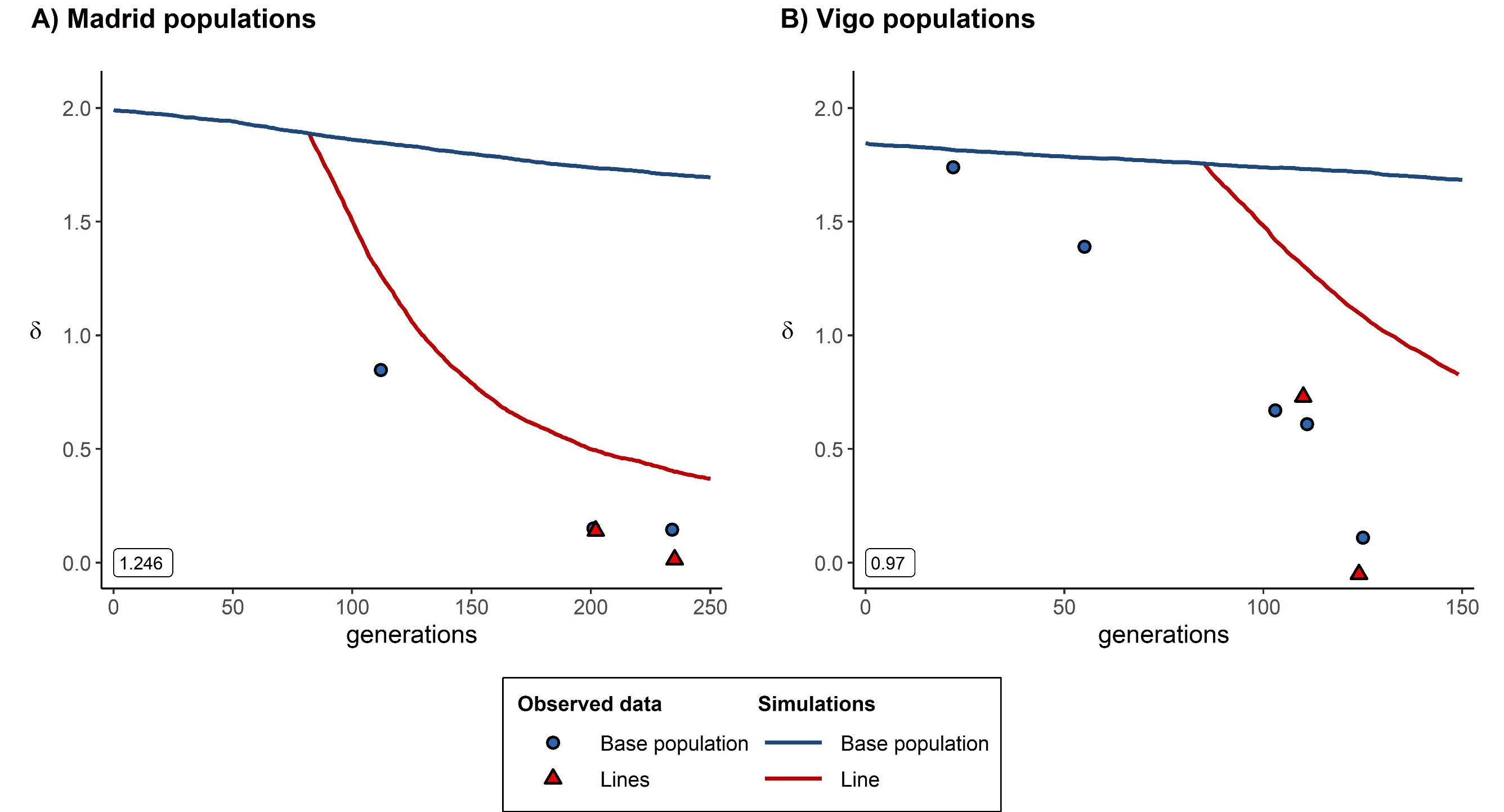


**Figure S5.** Evolution of relative fitness (*W*) corresponding to Madrid´s (A) and Vigo´s (B) populations. A simulated line of reduced size (*N* = 43 and 52 for panels A and B, respectively) derived from a base population (*N* = 1376 and 1000) after 83 and 86 generations, respectively, was run and values were averaged over 100 replicates. The mutational model was that assumed by Kyriazis et al. (2020), as explained in Figure S4 legend. Observed values (symbols) from Figure 2 of the main text are shown as a reference. The fit between simulation and predicted values is shown as the mean square difference (box) considering the values of the base population and small lines.


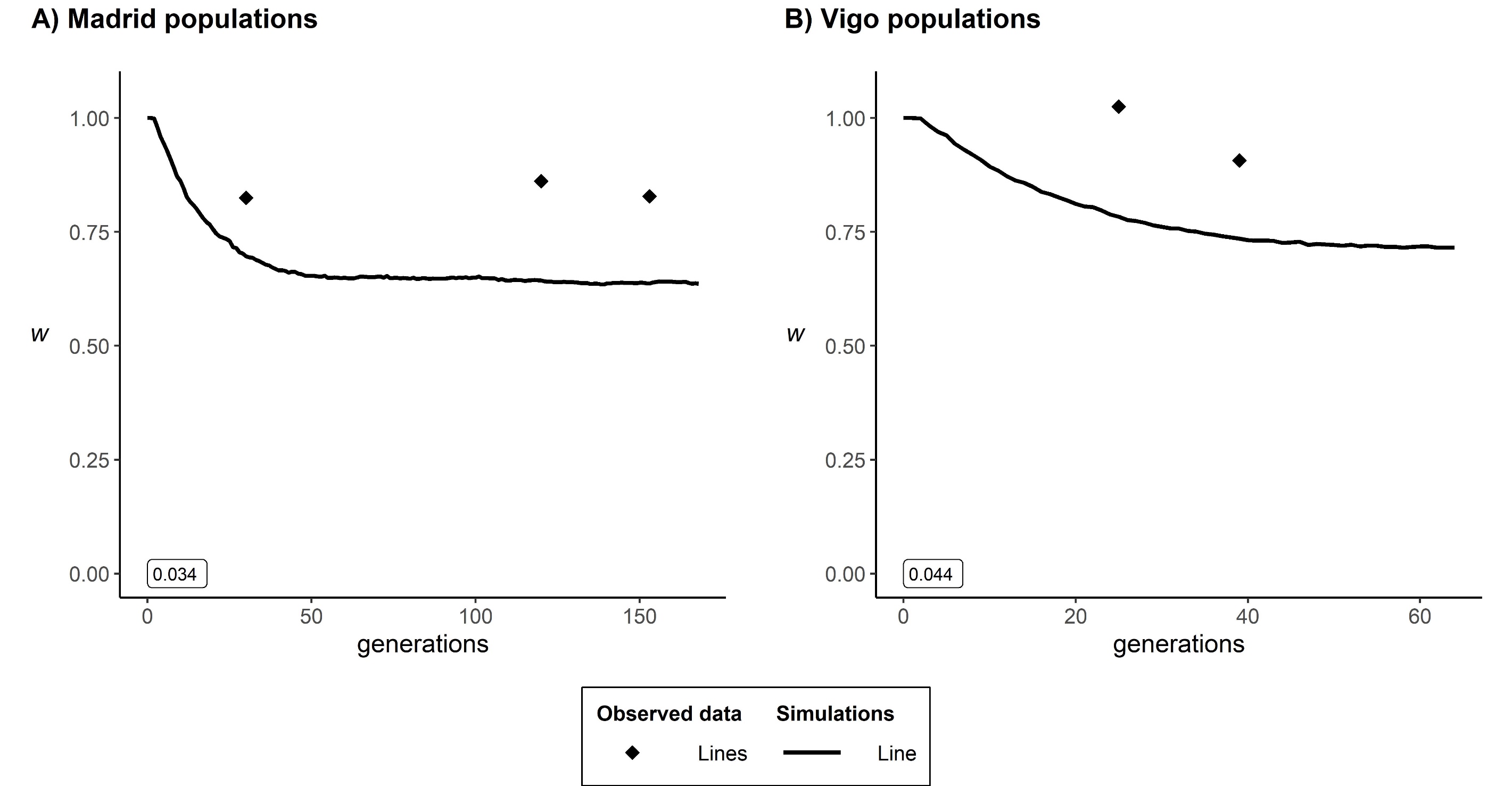


**Figure S6.** Evolution of the inbreeding depression rate (*δ*) in simulated populations corresponding to Madrid´s (A) and Vigo´s (B) populations. The simulated results (dark continuous lines) refer to those from Figure 3 of the main text for $\bar{s}$ = 0.2, $\bar{h}$ = 0.25 under a neutral model, i.e. only genetic drift is considered. Theoretical predictions (blue and red transparent lines) from Figure 1 of the main text are shown as a reference.


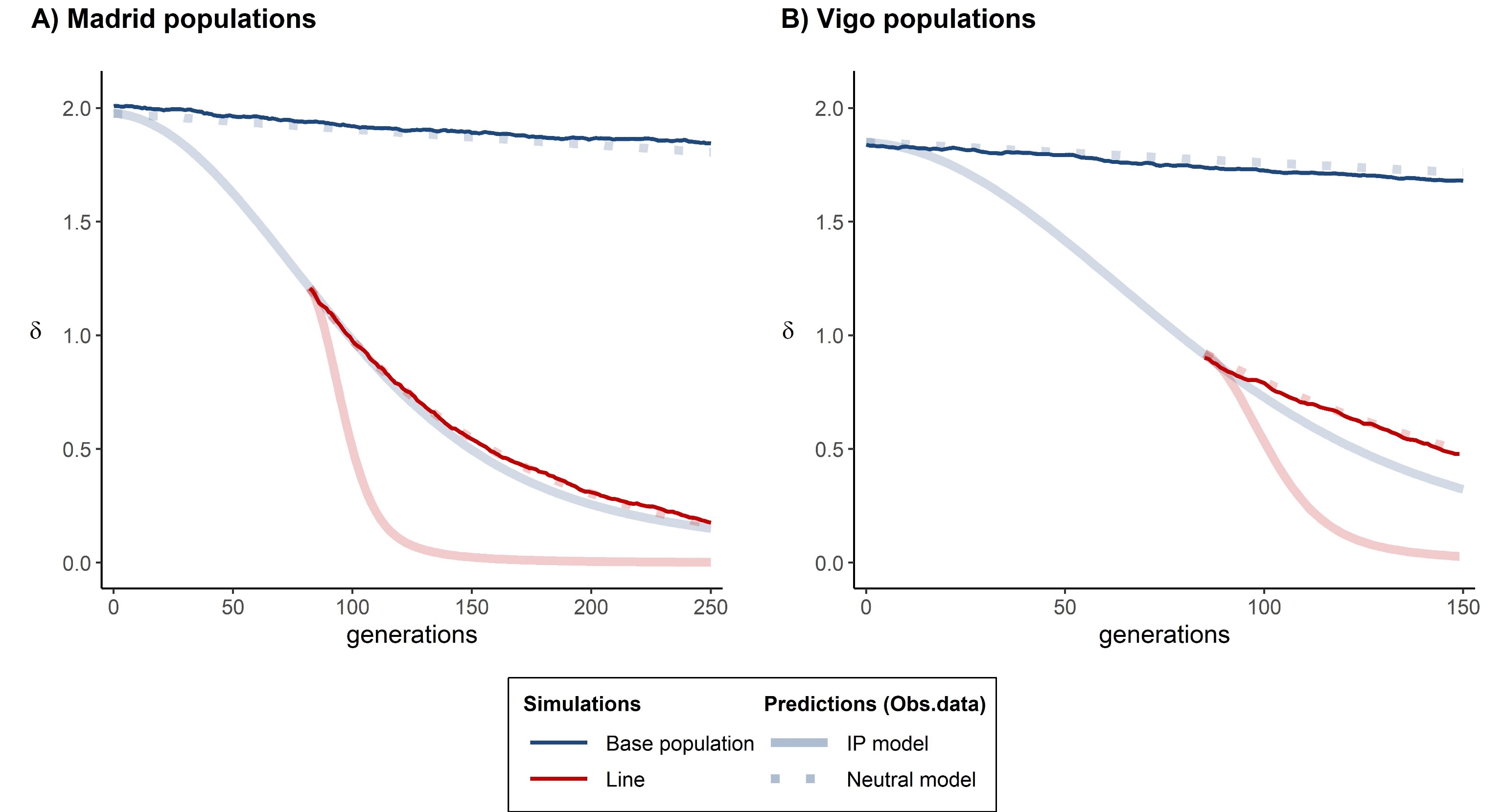


**Figure S7.** Evolution of relative fitness (*W*) corresponding to Madrid´s (A) and Vigo´s (B) populations. The simulated results (dark continuous lines) refer to those from Figure 4 of the main text for $\bar{s}$ = 0.2, $\bar{h}$ = 0.25 under a neutral model, i.e. only genetic drift is considered. Theoretical predictions (grey transparent lines) from Figure 2 of the main text are shown as a reference.


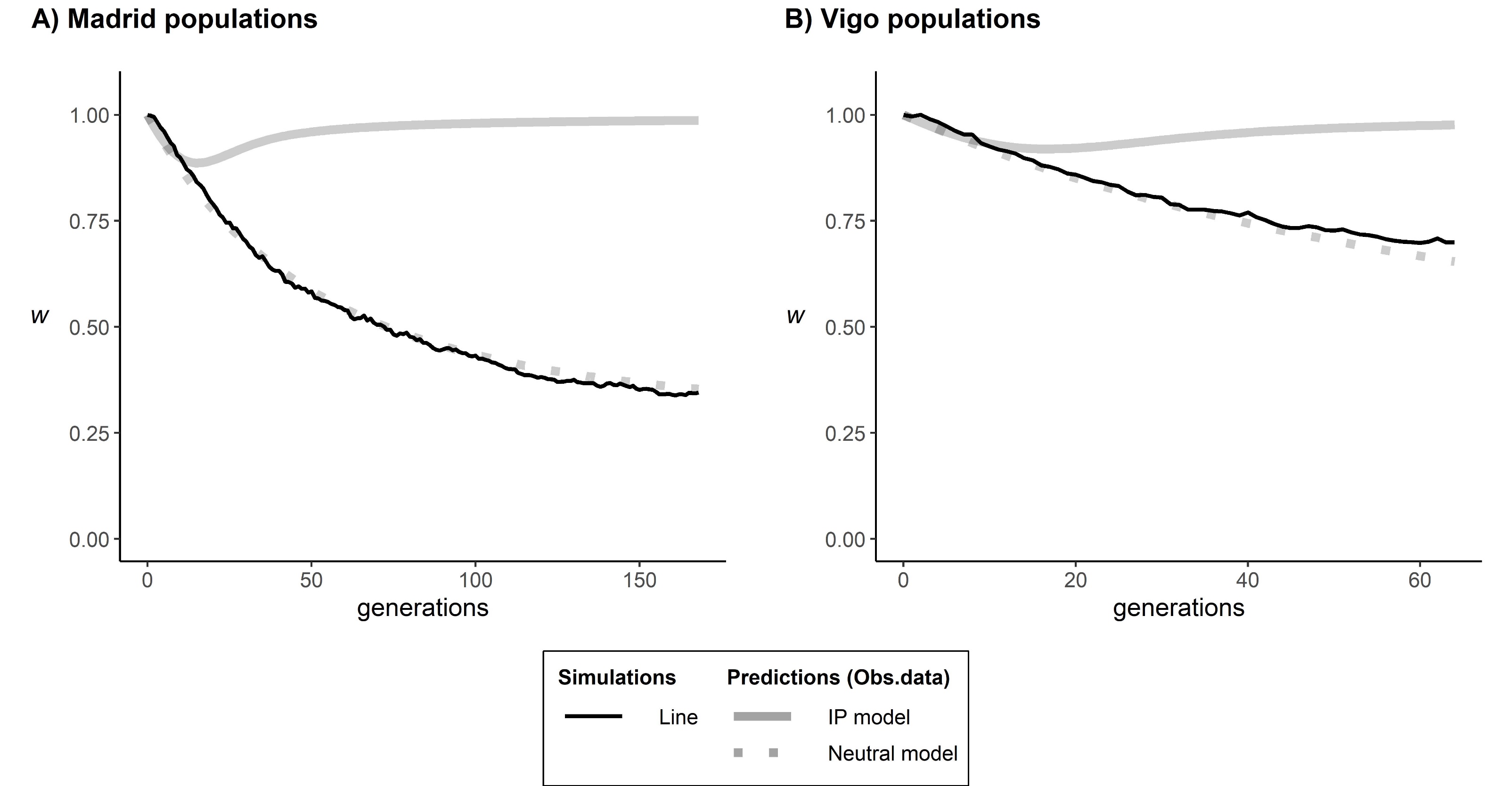

Supplement: Supplementary file 1 — Supplemental material [file 41437_2021_464_MOESM1_ESM.docx]
